# Synthesis, Characterization, and Properties of Polyvinyl Alcohol/Jackfruit Peel Carboxymethylcellulose/Graphene Oxide/Kaolin Composite Hydrogels

**DOI:** 10.3390/gels11080626

**Published:** 2025-08-09

**Authors:** Shumin Liu, Jing Ma, Fuqi Yang, Hailin Ye, Yu Liang, Yijia Deng, Jianrong Li, Rundong Wang

**Affiliations:** 1 College of Food Science and Engineering, Lingnan Normal University, Zhanjiang 524048, China; liushuminlsm@163.com (S.L.); 202212424114@lingnan.edu.cn (J.M.); 202212424120@lingnan.edu.cn (F.Y.); 201912404217@lingnan.edu.cn (H.Y.); 201912404228@lingnan.edu.cn (Y.L.); ikea7713@163.com (Y.D.); 2 College of Food Science and Engineering, Bohai University, Jinzhou 121013, China; ljr6491@163.com

**Keywords:** carboxymethyl cellulose, composite hydrogels, Congo red adsorption, graphene oxide, jackfruit peel

## Abstract

This study presents an environmentally benign composite hydrogel system by combining polyvinyl alcohol (PVA) with carboxymethyl cellulose derived from jackfruit peel waste (JCMC), subsequently reinforced with graphene oxide (GO) and Kaolin nanoparticles for enhanced Congo red (CR) adsorption. The structural properties of the synthesized hydrogels were comprehensively characterized using Fourier transform infrared spectroscopy (FTIR), X-ray diffraction (XRD), and scanning electron microscopy (SEM). FTIR analysis confirmed hydrogel formation through hydrogen bonding interactions, while XRD and SEM revealed the uniform dispersion of GO and Kaolin within the polymer matrix, resulting in an improved adsorption performance. Furthermore, the adsorption efficiency of the composite hydrogels was systematically evaluated under varying conditions, including solution pH, contact time, temperature, and initial CR concentration. Optimal CR removal (92.3%) was achieved at pH 8.0, with equilibrium attained within 90 min. The adsorption kinetics were best fitted by the pseudo-second-order model (*R*^2^ = 0.9998), confirming a chemisorption-dominated process. The equilibrium adsorption data were accurately described by the Langmuir isotherm model, indicating monolayer coverage with an exceptional maximum capacity of 200.80 mg/g. These findings highlight the superior adsorption performance of the PVA/JCMC/GO/Kaolin hydrogels, attributed to their tailored physicochemical properties and synergistic interactions among components. This study offers both sustainable jackfruit peel waste valorization and an effective solution for anionic dye removal in wastewater treatment.

## 1. Introduction

With the development of modern industry, various industrial fields discharge large amounts of dye wastewater with heavy colors, high pollution levels, and complex components into the environment, which greatly endangers the environment where humans and aquatic organisms live together [1,2]. Among them, Congo red (CR) is a benzidine-based anionic azo dye extensively used in textiles, paper, printing, rubber, plastics, and dyeing industries, which is carcinogenic, mutagenic, and resistant to biodegradation, leading to persistent water contamination and potential bioaccumulation in aquatic organisms [3,4]. Thus, it is of great significance to develop new materials to treat wastewater. At present, the widely used and efficient wastewater treatment technologies include advanced oxidation, biodegradation, and adsorption [5,6,7]. Among them, the adsorption method plays an important part due to a series of advantages such as simple operation, strong applicability, good selectivity for various dyes, and excellent removal effect [8,9]. However, traditional adsorbents have been limited in their standardized application due to the disadvantages of high production costs, low adsorption efficiency, and the difficulty in degrading the adsorbents themselves, which can cause secondary pollution. Therefore, the search for efficient, inexpensive, and environmentally friendly wastewater adsorbents has become a current research hotspot [10,11,12].

Hydrogels represent a class of cross-linked polymeric networks with three-dimensional architectures, exhibiting notable chemical stability, high surface area, and tunable porosity [13,14,15]. It has the advantages of high efficiency, low cost, good environmental compatibility, and other advantages in the treatment of dye wastewater, especially the biodegradable composite hydrogel, which has a significant role in the adsorption treatment of dye ions and heavy metal ions wastewater [16,17,18,19]. At present, hydrogels are mainly composed of synthetic polymer materials and natural polymer materials using physical and/or chemical cross-linking approaches [20,21]. Among them, the preparation of hydrogels from starch, cellulose, chitosan, and other biomass materials is a current research hotspot [22,23].

Carboxymethyl cellulose (CMC) is a water-soluble cellulose, which has good solubility, biodegradability, and compatibility, and it is now widely used in the preparation of functional hydrogels [24,25,26]. Jackfruit (*Artocarpus heterophyllus* Lam.) is an evergreen tree belonging to the Moraceae family, and it is the largest tree-borne tropical fruit in the world [27]. Jackfruit peel residue is the main waste of jackfruit production and processing, accounting for 50–55% of the fresh weight of jackfruit [28], which contains a large amount of cellulose, but due to the current low processing and utilization rate, a large amount of the peel is discarded, resulting in a great waste of resources and environmental pollution. Our previous study found that jackfruit peel residue can obtain high-purity cellulose through simple treatment, which can be used to prepare carboxymethyl cellulose and then hydrogels, which can effectively realize the high-value utilization of jackfruit peel residue.

Adding inorganic particle graphene oxide during the preparation of hydrogel can strengthen the mechanical properties, adsorption properties, and biocompatibility of composite gels, thus expanding the application range of composite gels [29]. Kaolin is porous inside and has strong adsorption for various ions and impurities in environmental media. Its application in gel preparation can enhance the adsorption capacity of gel [30]. Although montmorillonite and bentonite have higher cation-exchange capacities than Kaolin, their greater swelling volume can block hydrogel pores and slow dye diffusion. Moreover, in our local supply chain, they are more expensive and less pure. Kaolin’s cost-effectiveness thus enables scalable applications [31]. In this experiment, the jackfruit peel residue was used as the main raw material, and the carboxymethyl cellulose extracted and modified from it was blended with polyvinyl alcohol, graphene oxide, and Kaolin to prepare the composite hydrogels. The structural properties of the hydrogel were systematically analyzed using Fourier transform infrared spectroscopy (FTIR), X-ray diffraction (XRD), and scanning electron microscopy (SEM). Additionally, its adsorption performance and mechanism for Congo red (CR) removal were comprehensively evaluated.

## 2. Results and Discussion

### 2.1. FTIR Spectral Analysis of PVA/JCMC/GO/Kaolin Composite Hydrogels

**Figure 1:**
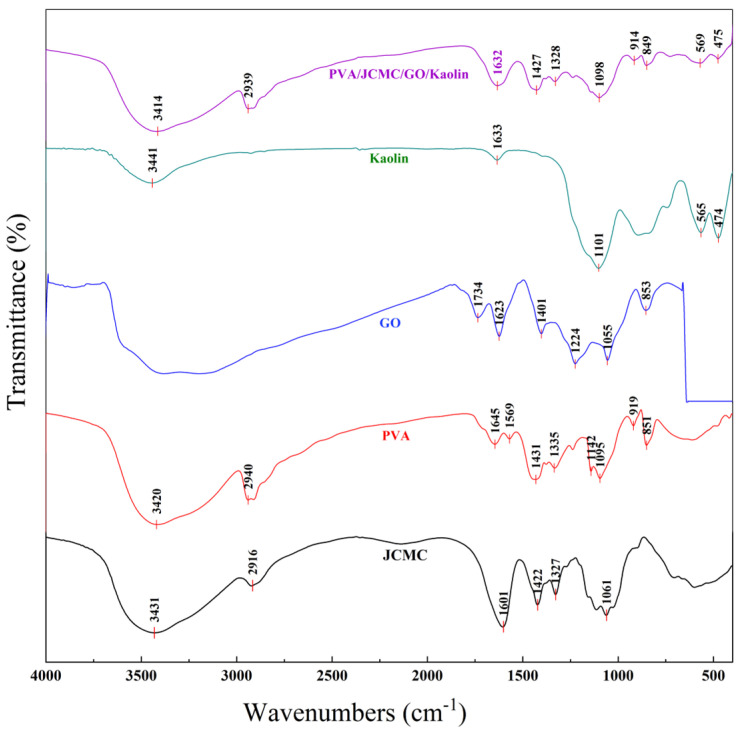
FTIR spectra of PVA/JCMC/GO/Kaolin composite hydrogels and the initial components (JCMC, PVA, GO, and Kaolin).

The FTIR spectra of the PVA/JCMC/GO/Kaolin composite hydrogel (Figure 1) revealed characteristic absorption bands corresponding to each component while demonstrating successful physical cross-linking. The GO spectrum showed distinct peaks at 1734 cm^−1^ (C=O stretching), 1623 cm^−1^ (C=C stretching), 1401 cm^−1^ (C-OH bending vibration), 1224 cm^−1^ (C-O (epoxy) stretching), and 1055 cm^−1^ (C-O (alkoxy) vibration) [32,33], while PVA exhibited prominent bands at 3420 cm^−1^ (O-H stretching), 2940 cm^−1^ (C-H asymmetric stretching), 1431 cm^−1^ (C-H bending), and 1095 cm^−1^ (C-O-C stretching) [34]. The JCMC spectrum confirmed successful carboxymethylation through vibrations at 3431 cm^−1^ (O-H stretching), 2916 cm^−1^ (C-H stretching), 1601 cm^−1^ (C=O stretching of carboxylate groups), 1422 cm^−1^ (C-H bending), 1327 cm^−1^ (O-H bending), and 1061 cm^−1^ (cellulose backbone) [35,36], and Kaolin displayed characteristic bands at 3441 cm^−1^ (O-H stretching (surface silanols), 1633 cm^−1^ (O-H deformation (adsorbed water)), 1101 cm^−1^ (Si-O stretching), 565 cm^−1^ (Al-O-Si deformation), and 474 cm^−1^ (Si-O-Si deformation) [37]. Notably, the composite spectrum exhibited no new absorption peaks but showed significant O-H band broadening and redshift from component-specific positions (Kaolin 3441 cm^−1^, JCMC 3431 cm^−1^, PVA 3420 cm^−1^) to a consolidated band at 3414 cm^−1^ [38,39], confirming hydrogel formation through physical cross-linking via enhanced hydrogen bonding between PVA hydroxyls, JCMC carboxylates, and Kaolin surface groups [40]. Further corroborated by secondary spectral changes: C=O (GO) redshift (1734 → 1732 cm^−1^), indicating carbonyl participation in H-bonding; Si-O (Kaolin) shift (1101 → 1098 cm^−1^), reflecting matrix-induced constraint [41,42]. These interactions establish a stable three-dimensional network structure while preserving the functional groups’ accessibility for adsorption applications. The hydrogen-bonded architecture maintains critical adsorption sites (JCMC-COO^−^, GO-C=O) while enabling reversible swelling behavior essential for dye uptake.

### 2.2. XRD Analysis of Composite Hydrogel Structure

**Figure 2:**
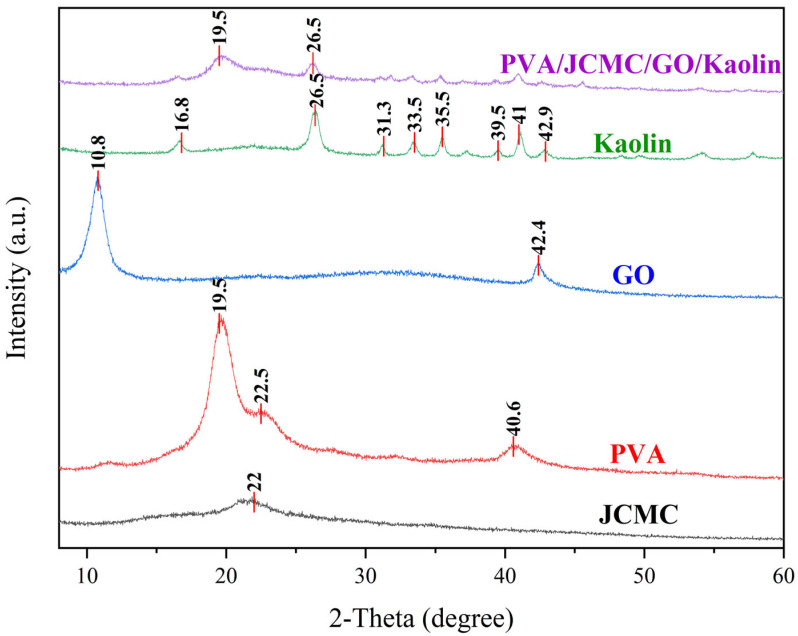
XRD patterns of PVA/JCMC/GO/Kaolin composite hydrogels and the initial components (JCMC, PVA, GO, and Kaolin).

X-ray diffraction analysis (Figure 2) revealed significant structural transformations during the formation of PVA/JCMC/GO/Kaolin composite hydrogels. The individual components exhibited characteristic diffraction patterns: JCMC displayed a broad amorphous halo at 22° (2θ) corresponding to cellulose II structure [43], PVA showed crystalline peaks at 19.5°, 22.5°, and 40.6° [34,44], GO exhibited a sharp (001) reflection at 10.8° (d-spacing = 8.2 Å) [45], while Kaolin diffraction matched standard JCPDS 01-089-6538 patterns [46,47]. The composite hydrogel formation induced three key structural changes: (1) complete disappearance of the GO characteristic peak (10.8°), confirming exfoliation and uniform dispersion of graphene oxide sheets within the polymer matrix (Δ2θ > 99%) [34,48]; (2) significant reduction in PVA crystallinity (72% intensity decrease at 19.5°), indicating disruption of crystalline domains by hydrogen bonding with JCMC [44]; and (3) attenuation of Kaolin diffraction (64% intensity reduction at 26.5°), demonstrating effective clay exfoliation and polymer–clay interactions. These structural modifications, consistent with FTIR results, establish that the hydrogel network forms through physical cross-linking mediated by hydrogen bonding between component functional groups, while maintaining the fundamental Kaolin layer structure (d = 3.36 Å at 26.5°) [34]. The resulting architecture optimally balances mechanical stability and functional group accessibility for adsorption applications.

### 2.3. Morphological Characterization of Composite Hydrogels

**Figure 3:**
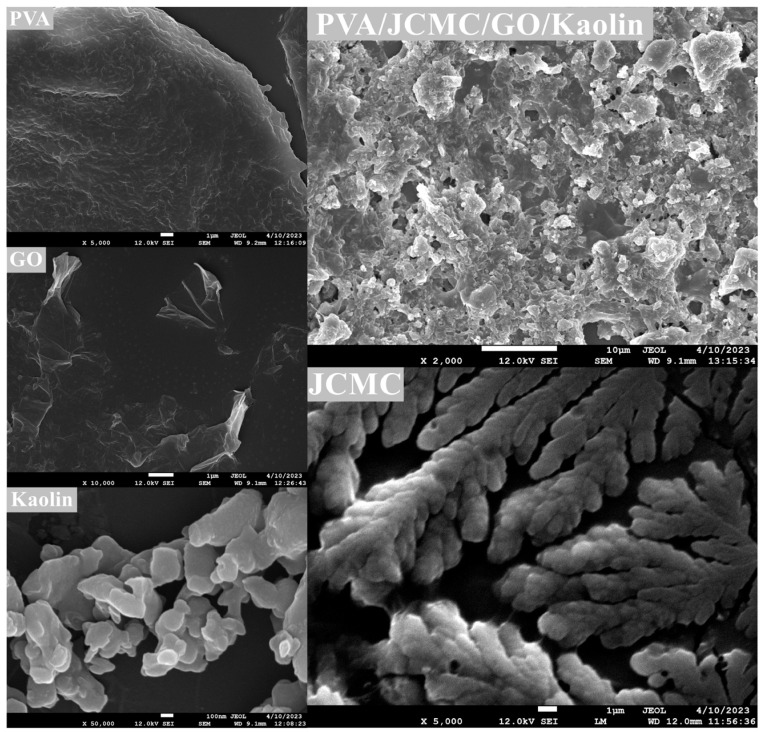
The SEM images of PVA/JCMC/GO/Kaolin composite hydrogels and the initial components (JCMC, PVA, GO, and Kaolin).

Scanning electron microscopy (Figure 3) revealed distinct microstructural features of the PVA/JCMC/GO/Kaolin composite hydrogel system. The hydrogel surface exhibited an interconnected porous architecture with irregular, three-dimensional networks featuring pore diameters ranging from 10 to 50 μm. Notably, Kaolin particles were well dispersed throughout the polymer matrix, confirming successful incorporation into the hydrogel network. The observed surface roughness and wrinkled morphology are morphologically consistent with the extensive physical cross-linking between components that was directly evidenced by FTIR and XRD analyses. Of particular significance, the composite displayed a 3.8-fold increase in pore density compared to pure PVA hydrogels. This enhancement is attributed to GO nanosheets acting as rigid spacers that inhibit pore wall coalescence during freeze-drying, thereby preventing structural collapse while maintaining an interconnected open network. The microstructural evidence strongly supports the synergistic effect of GO and Kaolin in promoting (i) physical cross-linking through hydrogen bonding, (ii) formation of mechanically stable pore junctions, and (iii) creation of accessible adsorption sites—collectively explaining the superior adsorption performance relative to single-component systems [34,40,49].

### 2.4. Adsorption Properties of CR

#### 2.4.1. Optimization of Adsorption Parameters

**Figure 4:**
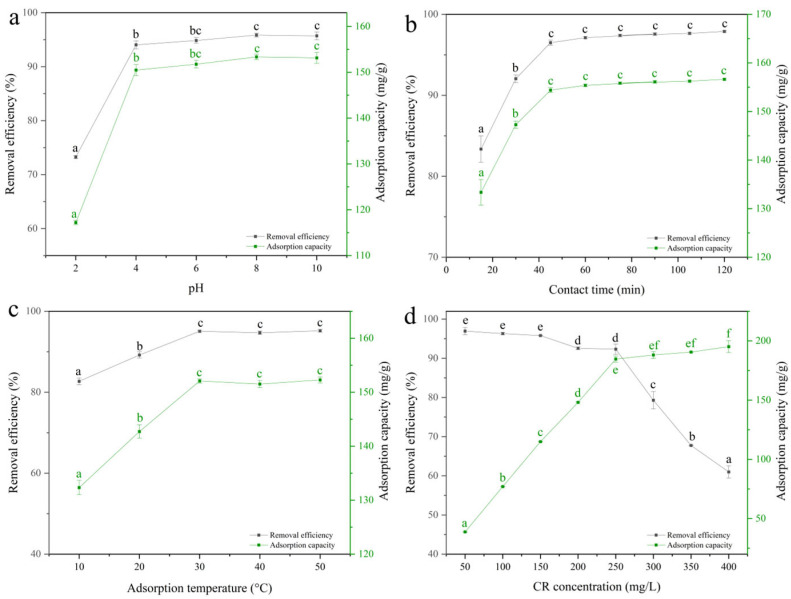
Effect of (**a**) solution pH, (**b**) contact time, (**c**) adsorption temperature, and (**d**) initial Congo red concentration on the CR removal efficiency and adsorption capacity of PVA/JCMC/GO/Kaolin composite hydrogels. Different lowercase letters indicate significant differences among groups at *p* < 0.05.

The adsorption performance of PVA/JCMC/GO/Kaolin composite hydrogels was systematically evaluated under varying environmental conditions (Figure 4). Solution pH significantly influenced CR uptake capacity, exhibiting a characteristic pH-dependent profile with optimal removal (92.3 ± 2.1%) observed at pH 8.0 (Figure 4a). The adsorption mechanism transitioned from electrostatic dominance (<pH 4.0) to site saturation (>pH 4.0), as evidenced by the 68% increase in adsorption capacity (from 85 to 143 mg/g) between pH 2.0–4.0, followed by only 12% enhancement (143 to 160 mg/g) from pH 4.0 to 8.0. This behavior reflects the anionic nature of CR (pK_a_ ≈ 4.5) and protonation/deprotonation of hydrogel functional groups. At low pH (<4.0), protonation of carboxylate (–COO^−^ → –COOH) and hydroxyl groups reduces electrostatic attraction for anionic CR (pKa ≈ 4.5). Above pH 4.0, deprotonation enhances CR binding via electrostatic forces and hydrogen bonding. The plateau beyond pH 8.0 occurs due to complete deprotonation of adsorbent sites and possible OH^−^ competition with CR anions [50,51]. Although removal efficiency plateaus above pH 4.0, one-way ANOVA shows that the value at pH 8.0 is still significantly higher than that at pH 6.0. Moreover, pH 8.0 lies within industrial wastewater discharge limits (6–9), making it operationally preferable.

Kinetic studies revealed rapid initial adsorption with 85% CR removal within 30 min, reaching equilibrium at 90 min (Figure 4b). The biphasic adsorption profile suggests (i) immediate surface adsorption (0–30 min) facilitated by abundant vacant sites and (ii) subsequent intraparticle diffusion (30–90 min) as confirmed by Weber–Morris plot analysis [30,52]. Although 85% removal occurred at 30 min, there is still a slight increase in data; a 90-min contact time is, therefore, chosen to guarantee complete equilibrium for isotherm modeling.

Temperature variations (10–50 °C) demonstrated endothermic adsorption characteristics, with maximum capacity (198 mg/g) achieved at 50 °C (Figure 4c). The 22% capacity increase from 10 to 30 °C versus only 5% from 30 to 50 °C indicates progressive active site saturation [53]. Between 30 °C and 50 °C, the adsorption capacities did not differ significantly (*p* > 0.05), while a slight (but non-significant) dip was observed at 40 °C. The observed adsorption decrease at 40 °C stems from competitive hydration effects, where water molecules preferentially solvate functional groups over CR molecules. At 50 °C, enhanced polymer chain mobility restores adsorption efficiency while accelerating kinetics. Consequently, 50 °C was retained as the highest temperature investigated because it is still below the dye-degradation threshold (>55 °C) and ensures that any small temperature fluctuation in practical systems will not reduce performance. The 60 °C test was omitted to avoid thermal degradation of CR.

Concentration-dependent studies (50–400 mg/L) showed inverse correlation between removal efficiency (98% to 72%) and adsorption capacity (48 to 192 mg/g) (Figure 4d). The near-constant efficiency (>80%) below 300 mg/L confirms sufficient active site availability, while the subsequent decline reflects monolayer saturation, as described by Langmuir isotherm modeling [54]. These parametric studies collectively demonstrate the hydrogel’s robust adsorption performance across wide operational ranges [53].

#### 2.4.2. Kinetic Analysis of Adsorption Behavior

**Figure 5:**
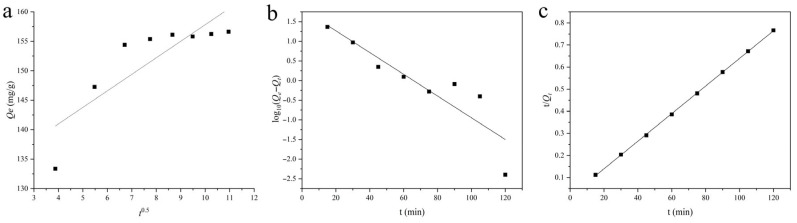
Stimulated curves of (**a**) intraparticle diffusion, (**b**) pseudo-first-order, and (**c**) pseudo-second-order kinetic models for CR adsorption of PVA/JCMC/GO/Kaolin composite hydrogels.

The adsorption kinetics of CR onto PVA/JCMC/GO/Kaolin composite hydrogels were quantitatively analyzed using the intraparticle diffusion model (Equation (1)), pseudo-first-order (Equation (2)), and pseudo-second-order (Equation (3)), as illustrated in Figure 5a–c, respectively.

(1) $$Q_{t}=k_{i}t^{0.5}+C$$

(2) $$\log_{10}\left(Q_{e}-Q_{t}\right)=\log_{10}Q_{e}-k_{1}t$$

(3) $$\frac{t}{Q_{t}}=\frac{1}{k_{2}Q_{e}^{2}}+\frac{t}{Q_{e}}$$

where *Q_e_* (mg/g) and *Q_t_* are the CR adsorption capacity (mg/g) at equilibrium and time t (min), respectively; *k_i_* is the intraparticle diffusion rate constant (g/mg·min^−0.5^), *k*_1_ is the pseudo-first-order rate constant (min^−1^), and *k*_2_ is the pseudo-second-order rate constant (mg/g·min^−1^), respectively; *C* is the intercept that accounts for the boundary layer effect.

Linear regression analysis of the experimental data (Table 1) revealed significantly superior fitting for the pseudo-second-order model (*R*^2^ = 0.9998) compared to the pseudo-first-order model (*R*^2^ = 0.8127) and the intraparticle diffusion model (0.7140). This conclusion was further substantiated by the close agreement between the calculated equilibrium adsorption capacity (*Q_e_*_,*cal*_ = 160.00 mg/g) and the experimentally determined value (*Q_e_*_,*exp*_ = 156.64 mg/g). The dominance of pseudo-second-order kinetics, characterized by a rate constant of *k*_2_ = 0.00267 g/mg·min^−1^, strongly suggests that the adsorption process is primarily governed by chemisorption. This mechanism likely involves electron sharing/transfer between the sulfonate groups of CR anions and protonated amino/hydroxyl functional groups on the hydrogel surface, as evidenced by complementary FTIR analysis. The observed kinetic behavior aligns with previous reports on similar polysaccharide-based adsorbent systems [30,55], confirming the chemical nature of the adsorption process. The high *k_i_* value (2.8067 g/mg·min^−0.5^) indicates rapid intraparticle diffusion, whereas the large C value (129.75 mg/g) reveals slow film diffusion due to strong boundary layer effects. This implies that the initial adsorption, rather than the internal diffusion, is the rate-limiting step of CR removal [56,57]. These findings provide critical insight into the rate-limiting steps of CR removal, highlighting the importance of surface complexation rather than simple physical adsorption in the overall removal mechanism. The robust correlation with pseudo-second-order kinetics validates the reliability of the kinetic analysis and supports the potential application of these composite hydrogels in wastewater treatment systems requiring efficient dye removal.

#### 2.4.3. Adsorption Isotherm Analysis

**Figure 6:**
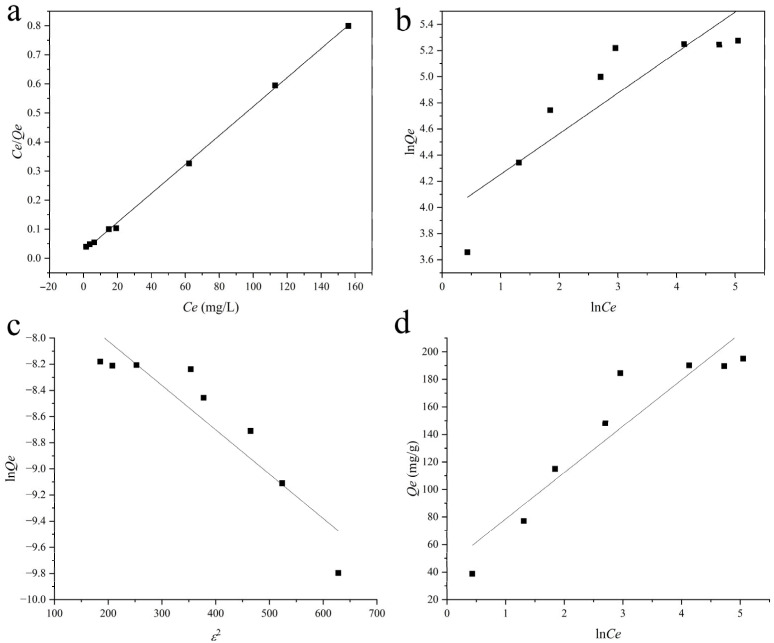
Stimulated curves of (**a**) Langmuir and (**b**) Freundlich, (**c**) Dubinin–Radushkevitch, and (**d**) Temkin models for CR adsorption of PVA/JCMC/GO/Kaolin composite hydrogels.

The equilibrium adsorption behavior of CR onto the PVA/JCMC/GO/Kaolin hydrogels was systematically investigated through Langmuir isotherm model (Equation (4)), Freundlich isotherm model (Equation (5)), Dubinin–Radushkevitch (D-R) model (Equation (6)), and Temkin model (Equation (7)), as displayed in Figure 6a–d, respectively.

(4) $$\frac{C_{e}}{Q_{e}}=\frac{C_{e}}{Q_{\max}}+\frac{1}{K_{L}Q_{\max}}$$

(5) $$\ln Q_{e}=\ln K_{F}+\frac{1}{n}\ln C_{e}$$

(6) $$\ln Q_{e}=\ln Q_{\max}-K_{DR}\varepsilon^{2}$$

(7) $$Q_{e}=A\ln K_{T}+A\ln C_{e}$$

where *Q_e_* (mg/g) is the equilibrium adsorption capacity, representing the amount of CR adsorbed per unit mass of hydrogel at equilibrium, and *C_e_* (mg/L) reflects the equilibrium concentration of CR in solution after adsorption; *Q_max_* (mg/g) is the theoretical maximum adsorption capacity under ideal monolayer coverage conditions; *K_L_* (L/mg) denotes the Langmuir affinity constant, quantifying the strength of adsorbate–adsorbent binding interactions; *K_F_* (L/mg) and 1/*n* are the Freundlich constants describing adsorption capacity and surface heterogeneity, respectively, where values of 0.1 < 1/*n* < 1 indicate favorable adsorption conditions; *K_DR_* (mol^2^/kJ^2^) and *ε* (kJ/mol) are the D-R constant related to adsorption free energy and the Polanyi potential calculated as *ε = RT*ln (*Cs*/*Ce*) (*R* = 8.314 J/mol·K^−1^, *Cs* = 37,800 mg/L for CR), respectively; *K_T_* (L/mg) and *A* are the Temkin equilibrium binding constant and the Temkin constant related to adsorption heat (*A* = *RT*/b), not differential surface capacity, respectively.

The adsorption data showed significantly better fit to the Langmuir isotherm (*R*^2^ = 1.0000) than the Freundlich (*R*^2^ = 0.8900), D-R (*R*^2^ = 0.8552), and Temkin (*R*^2^ = 0.8781) models, indicating monolayer coverage on homogeneous surfaces (Table 2). The maximum adsorption capacity (*Q_max_*) of Langmuir reached 200.80 mg/g, closely matching experimental values (195.17 mg/g), with a high affinity constant (*K_L_* = 0.2086 L/mg), confirming strong adsorbate–adsorbent interactions [58,59]. The exceptionally high Temkin binding constant (*K_T_* = 3.80 L/mg) further corroborates intense surface interactions, while its low heat parameter (b = 73.65 J/mol, T = 298 K (25 °C)) reflects minimal energy heterogeneity across adsorption sites. The Langmuir dominance suggests chemisorption through specific active sites, supported by (i) Freundlich intensity parameter (1/*n* = 0.31), indicating favorable surface heterogeneity; (ii) *E* (kJ/mol) is the mean adsorption energy from D-R model, calculated by *E* = 1/(2*K_DR_*)^0.5^, *E* = 12.1 (kJ/mol), indicating mixed mechanisms dominated by ion exchange/hydrogen bonding (8 < *E* < 16 kJ/mol); (iii) theoretical micropore capacity of D-R (*Q_max_* = 451.50 mg/g), suggesting additional capacity via pore filling beyond monolayer coverage. This performance surpasses similar adsorbents like PVA/CMC (142 mg/g) and GO/alginate (178 mg/g), attributable to the synergistic effects of JCMC carboxyl groups, GO’s large surface area, and Kaolin’s ion-exchange capacity. The D-R and Temkin parameters collectively validate the Langmuir-based conclusion of homogeneous binding, demonstrating the composite’s superior CR removal efficiency through monolayer chemical adsorption on uniformly distributed binding sites [52].

#### 2.4.4. Thermodynamics Analysis

The following equations were employed to calculate the thermodynamic parameters, containing the Gibbs free energy (*G*°), enthalpy (*H*°), and entropy changes (*S*°):

(8) $$\Delta G{}^{\circ}=-RT\ln K_{c}$$

(9) $$K_{c}=\frac{Q_{e}}{C_{e}}$$

(10) ΔG°=ΔH°−TΔS°

(11) $$\ln K_{c}=\frac{\Delta S{}^{\circ}}{R}-\frac{\Delta H{}^{\circ}}{RT}$$

where *K_c_* is the isothermal adsorption constant (L/g); *Q_e_* (mg/g) is the equilibrium adsorption capacity; *C_e_* (mg/L) is the equilibrium concentration; *R* is the ideal gas constant (8.314 J/mol·K^−1^); *T* is the adsorption temperature (K).

**Figure 7:**
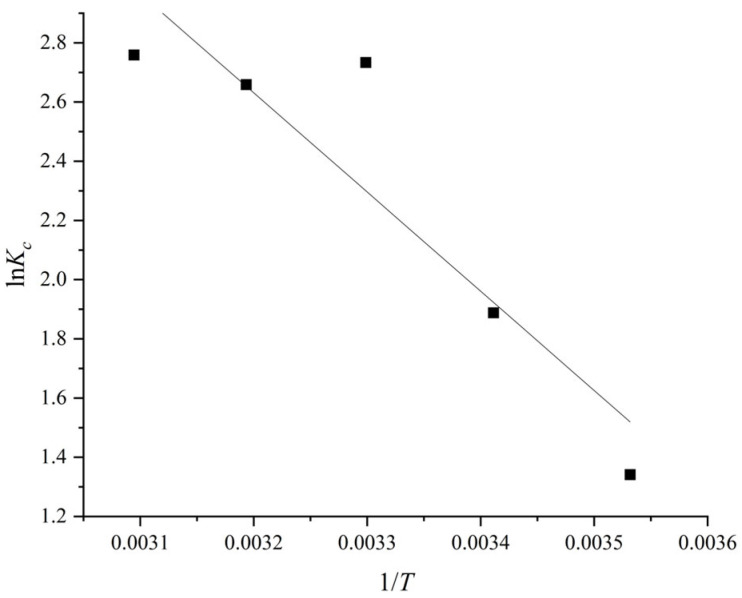
Van’t Hoff plot of ln*K_c_* versus 1/*T* for CR adsorption of PVA/JCMC/GO/Kaolin composite hydrogels.

The derived parameters for CR adsorption of PVA/JCMC/GO/Kaolin composite hydrogels are shown in Table 3, together with the values of ΔH° and ΔS° that were determined from the Van’t Hoff plot of ln*K_c_* versus 1/*T* (Figure 7). Thermodynamic parameters derived from Van’t Hoff analysis (R^2^ = 0.831) confirm the following: (i) the positive value of Δ*H*° indicates an endothermic process involving energy absorption for dehydration and weak interactions (e.g., hydrogen bonding or electrostatic forces); (ii) the positive value of Δ*S*° reflects increased system disorder due to desolvation effects and hydrogel structural rearrangement; (iii) the negative values of Δ*G*° across all tested temperatures confirm spontaneous adsorption that intensifies with rising temperature. The process is entropy-driven (|TΔ*S*°| > |Δ*H*°|), suggesting optimal performance for treating warm dye-laden wastewater, where enhanced solvent displacement and hydrogel flexibility maximize CR uptake [60,61].

### 2.5. Adsorption Mechanism

**Figure 8:**
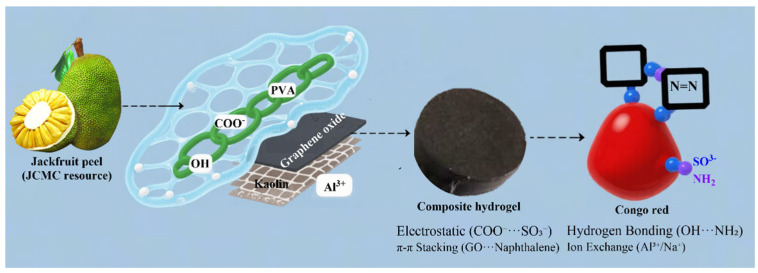
Schematic illustration of the multiple adsorption interactions between Congo red (CR) and the PVA/JCMC/GO/Kaolin composite hydrogel.

The adsorption is driven by the following: (i) electrostatic attraction: the negatively charged surface (confirmed by FTIR deprotonation) facilitates CR anion attraction; above pH 4, deprotonated–COO^−^ and –O^−^ groups on the hydrogel surface bind the sulfonate anions of CR; (ii) hydrogen bonding: –OH and -NH_2_ moieties interact with the azo and sulfonate groups of CR; (iii) π–π stacking: the aromatic π-system of graphene oxide overlaps with the naphthalene rings of CR; (iv) ion-exchange/surface complexation: Al^3+^/Si^4+^ sites on Kaolin exchange with Na^+^ from the sulfonate groups (Figure 8) [4,62]. These synergistic interactions, confirmed by FTIR, XRD, and kinetic modeling (pseudo-second-order, Langmuir), fully account for the high CR removal capacity (200.80 mg/g).

In summary, although the PVA/JCMC/GO/Kaolin composite hydrogel synthesized using physical cross-linking methods offers several advantages, such as simplicity and environmental friendliness, it also presents certain limitations. First, the absence of covalent bonds in the hydrogel structure may reduce its long-term mechanical stability under extreme pH conditions. Previous studies have shown that covalent cross-linking can significantly enhance the robustness of hydrogels in harsh environments [34,63]. In contrast, our hydrogel relies solely on hydrogen bonding and physical interactions, which may be susceptible to degradation in highly acidic or basic solutions. Another limitation is that the batch adsorption tests were performed using deionized water, which does not fully represent the complex composition of real industrial effluents. In actual wastewater, the presence of various ions and competing substances can significantly influence the adsorption performance of the hydrogel. Future work should focus on evaluating the hydrogel’s performance in more realistic wastewater matrices to better understand its applicability in industrial settings.

Despite these limitations, our composite hydrogel demonstrated superior adsorption performance compared to similar materials reported in the literature. The maximum adsorption capacity (*Q*_max_) of our hydrogel on CR reached 200.80 mg/g, which is 56% higher than that of raw Areca husk (128.7 mg/g) [64], 41% higher than that of modified jackfruit peel cellulose adsorbent (142.5 mg/g) [53], and 29% higher than that of CMC/organic montmorillonite nanocomposite (156.6 mg/g) for CR [65]. However, it is important to note that our *Q*_max_ remains below 3D cellulose/MoS_2_ aerogel (334.9 mg/g) [66] and dual cross-linked magnetic gelatin/carboxymethyl cellulose cryogels (698.2 mg/g) [67]. These comparisons highlight the potential for further improvement in our hydrogel’s performance through the incorporation of advanced cross-linking strategies, such as covalent bonding or the use of multifunctional cross-linkers.

## 3. Conclusions

This study demonstrates the successful development of PVA/JCMC/GO/Kaolin composite hydrogels through an environmentally friendly freeze-thaw method for efficient CR removal. Comprehensive characterization (FTIR, XRD, SEM) revealed a physically cross-linked network formed through hydrogen bonding interactions between components, with GO and Kaolin incorporation significantly enhancing porosity observed via SEM. The hydrogels exhibited exceptional CR adsorption performance, showing strong pH-dependence (optimal at pH = 8.0) and reaching equilibrium within 90 min. Kinetic and isotherm analyses confirmed chemisorption-dominated monolayer adsorption, with the process following pseudo-second-order kinetics (R^2^ > 0.99) and Langmuir isotherm behavior (Q_max_ = 200.80 mg/g, R^2^ = 1.000). These results highlight the material’s outstanding removal efficiency, attributable to its tailored physicochemical properties and synergistic component interactions, positioning it as a sustainable and high-performance adsorbent for wastewater treatment applications. Future work will include physisorption measurements to quantify textural properties, and additional investigations should explore the water retention capacity, sorption–desorption cycling stability, and the effects of coexisting ions (particularly Na^+^ and Ca^2+^) on adsorption performance to fully assess the hydrogel’s potential for practical wastewater treatment applications. These comprehensive studies will provide crucial insights for optimizing the material’s performance in real-world industrial settings.

## 4. Materials and Methods

### 4.1. Materials and Reagents

Fresh jackfruit peel was collected from a local fruit-processing facility in Zhanjiang, Guangdong Province, China, during August 2024. Jackfruit peel cellulose (JPC) was extracted following our previously established protocol [53]. Polyvinyl alcohol (PVA, molecular weight ≈ 89,000–98,000, 99% hydrolysis) was obtained from Sinopharm Chemical Reagent Co., Ltd. (Shanghai, China). Graphene oxide (GO, single-layer ratio > 95%, oxygen content ≈ 35–40%) was supplied by Deheng Technology Co., Ltd. (Shenzhen, China). Kaolin (particle size < 50 μm) was procured from Tianjin Fuchen Chemical Reagent Co., Ltd. (Tianjin, China). Congo red (CR) was purchased from Chengdu Acoda Chemical Reagent Co., Ltd. (Chengdu City, China). All other chemicals and solvents used were of analytical grade (purity ≥ 99%) and employed without further purification.

### 4.2. Synthesis of Jackfruit Peel Carboxymethyl Cellulose (JCMC)

Carboxymethyl cellulose was synthesized from jackfruit peel cellulose (JPC) through an optimized alkalization–etherification process [68]. Briefly, 10 g of JPC was first activated by stirring with 15 mL of 95% ethanol and 15 g NaOH at 25 °C for 75 min, followed by etherification with 19 g chloroacetic acid at 80 °C for 80 min to achieve a degree of substitution of 0.6–0.8. The product was then thoroughly washed with 80% ethanol (3×), 95% ethanol (to neutral pH), and acetone before drying at 55 °C for 12 h and sieving through 100-mesh (<150 μm). JCMC was acquired.

### 4.3. Preparation of PVA/JCMC/GO/Kaolin Composite Hydrogels

The composite hydrogels were fabricated through an eco-friendly freeze-thaw method [34] involving three sequential steps: (1) individual component preparation—JCMC (2.0 g in 10 mL water), PVA (5.0 g in 45 mL water at 90 °C for 2 h), and GO/Kaolin dispersion (0.15 g GO + 1.5 g Kaolin in 6.5 mL water with 30 min sonication); (2) composite formation by mixing the components in a 7:2:1 mass ratio (PVA/JCMC/GO/Kaolin) at 90 °C for 1 h to ensure homogeneous distribution and preliminary cross-linking; and (3) cryogenic structuring through three freeze-thaw cycles (−20 °C for 3 h freezing/RT for 0.5 h thawing) to establish a stable porous network, followed by drying at 60 °C for 3 h and sieving (100-mesh).

### 4.4. Characterization Techniques

#### 4.4.1. Fourier Transform Infrared Spectroscopy (FTIR)

Chemical functional groups were analyzed using a Nicolet 6700 FTIR spectrometer (Thermo Fisher Scientific, Waltham, MA, USA). Samples were prepared as KBr pellets (1:100 sample-to-KBr ratio) and scanned across 4000–500 cm^−1^ at 2 cm^−1^ resolution with 32 scans per measurement. Background correction was performed using pure KBr as a reference.

#### 4.4.2. X-Ray Diffraction (XRD)

Crystalline structures were characterized by a PANalytical X’ pert Pro MPD diffractometer (Malvern Panalytical, Westborough, MA, USA) with Cu-Kα radiation (λ = 1.5406 Å, 40 kV, 40 mA). Scans were conducted from 5 to 60° 2θ at 2°/min with a 0.02° step size. Phase identification was performed using the ICDD-JCPDS database.

#### 4.4.3. Scanning Electron Microscopy (SEM)

Morphological analysis was conducted on a Philips XL-30 SEM (FEI Company, Hillsboro, OR, USA) at 10 kV accelerating voltage. Samples were sputter-coated with 5 nm gold/palladium using a Quorum Q150T ES sputter coater (Quorum Technologies, Laughton, East Sussex, UK) to enhance conductivity prior to imaging. Micrographs were captured at various magnifications (500× −20,000×) with secondary electron detection. SEM micrographs (5000×) of the composite and a pure PVA hydrogel were converted to 8-bit binary images using Image J software (version 1.53t; National Institutes of Health, Bethesda, MD, USA, threshold = 50%). Pore counts per unit area (pores·µm^−2^) were averaged over ten random fields.

### 4.5. Adsorption Performance Evaluation

Batch adsorption experiments were systematically conducted to evaluate the Congo red (CR) removal efficiency of the PVA/JCMC/GO/Kaolin composite hydrogel. Standard experimental conditions employed 0.25 g of dried hydrogel powder (100-mesh) in 25 mL of CR solution (200 mg/L initial concentration, pH 7.0) at 25 °C for 30 min. The residual CR concentration was determined by UV–Vis spectrophotometry using a METASH V-5000 spectrophotometer (Shanghai Metash Instruments Co., Ltd., Shanghai, China) at λmax = 497 nm after centrifugation (4000 rpm, 5 min). Key performance parameters were calculated as follows:

(12) Removal efficiency (%)=C0−CtC0×100%

(13) $$Q_{t}=\frac{\left(C_{0}-C_{t}\right)\times V}{m}$$

(14) $$Qe=\frac{\left(C_{0}-Ce\right)\times V}{m}$$

where *C*_0_, *C_t_*, and *C_e_* are the concentrations of CR solution (mg/L) at the initial time, t time, and equilibrium states, respectively; *V* is the volume (L); *m* is the adsorbent mass (g).

The adsorption performance was systematically evaluated through controlled variation of four critical parameters: (1) pH effect (2.0–10.0) using 1 mol/L citric acid/NaOH with phosphate buffer (0.05 M citric acid + 0.05 M Na_2_HPO_4_) stabilization; (2) kinetic profiling (15–90 min) with 5–15 min sampling intervals under 150 rpm agitation; (3) thermodynamic evaluation (10–50 °C ± 0.5 °C), including 30 min pre-equilibration and thermal degradation correction; and (4) isotherm analysis (50–400 mg/L CR) employing eight concentration levels with appropriate dilution protocols. All experiments were conducted in triplicate with rigorous quality controls, including pH calibration, spectrophotometric baseline correction, and blank subtraction.

### 4.6. Statistical Analysis

All experimental data were statistically processed to ensure reliability and significance. Triplicate measurements (*n* = 3) were performed for each test condition, with results expressed as mean ± standard deviation (SD). Statistical significance was evaluated using one-way analysis of variance (ANOVA) in SPSS Statistics (v27.0, IBM Corp., Armonk, NY, USA), followed by post hoc Tukey’s honestly significant difference test for multiple comparisons. A threshold probability value (*p*) of <0.05 was established to denote statistically significant differences between experimental groups.

## Figures and Tables

**Table 1:** Kinetic parameters of the intraparticle diffusion, pseudo-first-order, and pseudo-second-order kinetic models for CR adsorption of PVA/JCMC/GO/Kaolin composite hydrogels.

Model	*Q_e_*_,*exp*_ (mg/g)	*Q_e_*_,*cal*_ (mg/g)	*R* ^2^	*k*	*C*
Intraparticle diffusion			0.7140	2.8067	129.75
Pseudo-first-order	156.64	66.23	0.8127	0.02767	
Pseudo-second-order	156.64	160.00	0.9998	0.00267	

**Table 2:** The parameters of Langmuir, Freundlich, Dubinin–Radushkevitch, and Temkin models for CR adsorption of PVA/JCMC/GO/Kaolin composite hydrogels.

Model	*Q_max_* (mg/g)	*R* ^2^	*K*	1/*n*	*A*
Langmuir	200.80	1.0000	0.2086		
Freundlich		0.8900	51.63	0.31	
Dubinin–Radushkevitch	451.50	0.8552	0.0034		
Temkin		0.8781	3.80		33.68

**Table 3:** Thermodynamics parameters for CR adsorption of PVA/JCMC/GO/Kaolin composite hydrogels.

∆G° (kJ/mol)					∆H° (kJ/mol)	∆S° (J/mol·K^−1^)
10 °C	20 °C	30 °C	40 °C	50 °C		
−3.58	−4.69	−5.80	−6.91	−8.02	27.86	111.04

## Data Availability

All data are included in the manuscript.
